# Design, Synthesis and Biological Evaluation of New Imidazo[2,1-b]Thiazole Derivatives as Selective COX-2 Inhibitors

**Published:** 2018

**Authors:** Mahsa Shahrasbi, Mahsa Azami Movahed, Orkideh Ghorban Dadras, Bahram Daraei, Afshin Zarghi

**Affiliations:** a *Department of Medicinal Chemistry, Pharmaceutical Sciences Branch, Islamic Azad University, Tehran, Iran. *; b *Department of Pharmaceutical Chemistry, School of Pharmacy, Shahid Beheshti University of Medical Sciences, Tehran, Iran. *; c *Department of Toxicology, School of Pharmacy, Shahid Beheshti University of Medical Sciences, Tehran, Iran.*

**Keywords:** Design, Synthesis, Cyclooxygenase-2 inhibition, Imidazo[2, 1-b]Thiazole, Molecular modeling

## Abstract

A new series of imidazo[2,1-b]thiazole analogs containing a methyl sulfonyl COX-2 pharmacophore was synthesized and evaluated for their COX-2 inhibitory activity. According to *in-vitro* COX-1/COX-2 inhibition data, all compounds (6a-g) were selective inhibitors of COX-2 isoenzyme with IC_50_ values in the highly potent 0.08-0.16 µM range. These results indicated that both potency and selectivity of COX-2 inhibitory activity were affected by the type and size of amine on C-5 of imidazo[2,1-b]thiazole ring. Our data identified N,N-dimethyl-1-(6-(4-(methylsulfonyl)phenyl)imidazo[2,1-b]thiazol-5-yl)methanamine (6a) as a potent and selective COX-2 inhibitor (IC_50 _COX-1 >100 µM; IC_50 _COX-2 = 0.08 µM; selectivity index = 313.7). Our results indicated that both potency and selectivity of COX-2 inhibitory activity were affected by the type and size of amine on C-5 of imidazo[2,1-b]thiazole ring.

## Introduction

**Figure 1:**
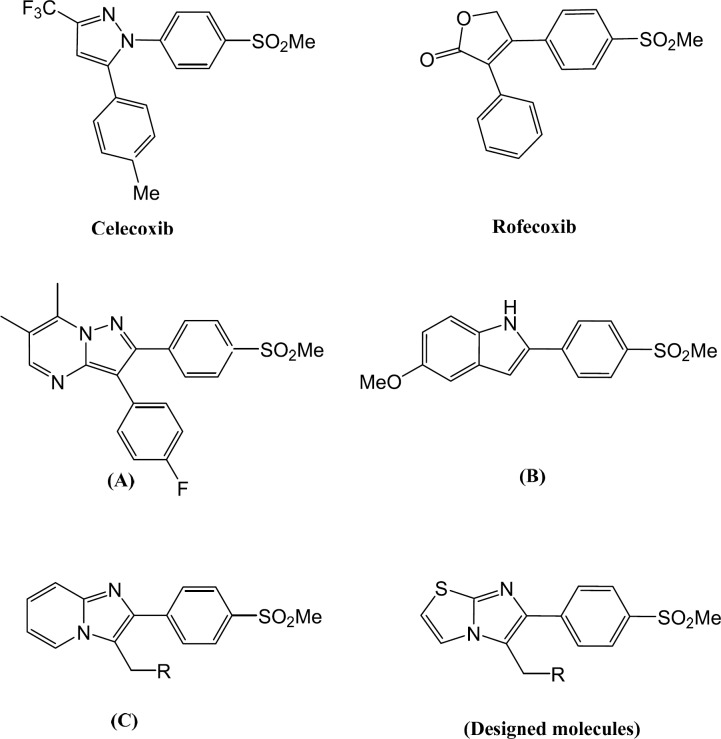
Chemical structures of COX-2 inhibitors (Celecoxib and Rofecoxib), lead compounds (A, B, C) and our designed molecules

Non-steroidal anti-inflammatory drugs (NSAIDs) can relieve inflammation, fever, and pain by inhibiting cyclooxygenase. Cyclooxygenase (COX) is the key enzyme in the biosynthesis of inflammation mediators, prostaglandins (1). There are three identified isozymes of COX. COX-1 which has physiological roles in the body is expressed in many tissues and shows important effects in homeostasis maintenance. COX-2 is the inducible isozyme and has roles in pathological conditions such as inflammation. In addition, recent studies reveal that COX-2 levels are elevated in some other diseases such as cancer (2). Another isoform of cyclooxygenase, COX-3 is responsible for pain and fever (3). NSIADs reduce pain, fever and inflammation by inhibition of both COX-1 and COX-2. COX-1 inhibitory activity of NSAIDs leads to some side effects like renal dysfunction and gastrointestinal ulcers (4, 5). Accordingly, it was expected that selective inhibition of COX-2 could reduce gastrointestinal side effects of NSAIDs while exhibiting desirable anti-inflammation effects of them. Therefore, many different selective COX-2 inhibitors have been synthesized and evaluated to have less gastrointestinal side effects than NSAIDs. Moreover, COX-2 expression increases in cancer (6-9), Parkinson’s (10) and Alzheimer′s disease (11). This fact theorizes that we can decrease the progression of these diseases by COX-2 inhibition. Based on this fact, many different structures of COX-2 inhibitors were investigated (12-23). Although their structures have very high varieties, we can classify them to two main classes of tricyclics and non-tricyclics (24). Tricyclics included the majority of COX-2 inhibitors. These compounds possess vicinal diaryl on a hetero/carbocyclic central ring and a pharmacophore group of methylsulfonyl, sulfonamide, or azido on *para* position of one of the aryl rings which is responsible for COX-2 selectivity such as well-known compounds Celecoxib (25), Rofecoxib (26) and Valdecoxib (27). Increased risk of cardiovascular events due to Rofecoxib use, led to voluntarily withdrawing of this drug from the market. Some studies demonstrated that cardiac adverse effects associated with the use of COX-2 inhibitors may be related to intrinsic chemical properties of the drugs not the class effects of these medications (28). Accordingly, introducing new COX-2 scaffolds with valuable biological activities and improved safety profiles is still one of the researcher′s interests. Almansa *et al.* introduced a series of pyrazolo[1,5-*a*]pyrimidine derivatives and studied addition of a fused ring to usual five-memberd central ring. Among this series, 3-(4-fluorophenyl)-6,7-dimethyl-2-(4-(methylsulfonyl) phenyl)pyrazolo[1,5-a]pyrimidine (Figure 1) exhibited the most potency and selectivity (IC_50 _Whole cell COX-2 = 0.012 µM, COX-1 > 10 µM) (29). We reported an investigation on 5-substituted-2-(4-(azido or methylsulfonyl)phenyl)-1*H*-indole derivatives (30). In this study, 5-methoxy-2-(4-(methylsulfonyl)phenyl)-1*H*-indole (Figure 1) was the most potent and selective compound among synthesized compounds (COX-2 IC_50_= 0.08 µM; SI = 291.2). Also, we have recently reported a group of imidazo[1,2-a]pyridine derivatives with remarkable potency and selectivity. In this series, the most potency and selectivity of COX-2 inhibition belongs to 2-(4-(methylsulfonyl)phenyl)-3-(morpholinomethyl)*H*-imidazo[1,2-a]pyridine (IC_50 _= 0.07 µM, SI = 217.1) (31). In present work, we changed pyridine ring in imidazo[1,2-a]pyridine to thiazole ring in imidazo[2,1-b]thiazole and we designed a new group of 6-(4-(methylsulfonyl)phenyl)imidazo[2,1-b]thiazole derivatives possessing a methylsulfonyl at *para *position of C-6 phenyl ring and different mannich base on C-5 (Figure 1).

## Experimental

*Materials and Methods*

All reagents used in this study were purchased from Merck AG and Aldrich Chemical companies without further purifications. Melting points were determined with a Thomas–Hoover capillary apparatus. Perkin Elmer Model 1420 spectrometer was used to acquire Infrared spectra. ^1^HNMR spectra with TMS as internal standard were acquired with Bruker FT-500 MHz instrument (Brucker Biosciences, USA. Chloroform-D was used as solvent. Coupling constant (J) values are estimated in hertz (Hz) and spin multiples are given as s (singlet), d (double), t (triplet), q (quartet), and m (multiplet). The mass spectral measurements were performed on a 6410 Agilent LCMS triple quadrupole mass spectrometer (LCMS) with an electrospray ionization (ESI) interface. Microanalyses, determined for C and H, were within ±0.4% of theoretical values. 

*Preparation of 4-(methylthio)acetophenone (* ***2*** *)*

11.5 g AlCl_3_ (87 mmol) was suspended in 200 mL CHCl_3 _and stirred until disperse uniformly. After cooling to 10 ºC, 6.2 mL (87 mmol) acetyl chloride was added, followed by dropwise addition of 7.5 mL (80 mmol) thioanisole when the temperature was under 4 ºC and allowed the mixture to stir at room temperature for 2 h. The mixture was poured into crushed ice, organic layer was separated with CHCl_3_ and washed with saturated NaHCO_3_ solution and finally dried over anhydrous Na_2_SO_4_. The solvent was evaporated under reduced pressure. The precipitate was filtered and washed with n-hexane. Yield 87%; white crystalline powder; mp: 81-82 ºC; IR (KBr disk): υ (cm^-1^) 1690 (C=O); LC-MS (ESI) m/z: 167 (M+1, 100).

*4-(Methylsulfonyl)acetophenone (* ***3*** *)*

To a solution of 4 g of 1 (24 mmol) in 20 mL THF, mixture of 20 g oxone in THF and water (1:1) was added and stirred at room temperature for 2 h. THF was removed under reduced pressure and extracted with CHCl_3_ (3 × 25 mL). The organic layer was washed twice with saturated NaHCO_3_ solution and dried over anhydrous Na_2_SO_4_. The solvent was evaporated and white precipitate was recrystallized in ethanol. Yield 95%; white crystalline powder; mp: 128-130 ºC; IR (KBr disk): υ (cm^-1^) 1148, 1309 (SO_2_), 1681 (C=O); LC-MS (ESI) m/z: 198.9 (M+1, 100).

*α-Bromo-4-(methylsulfonyl)acetophenone (* ***4*** *)*

Dissolve 2 g (10.1 mmol) of 2 in 20 mL CHCl_3_. The bromine was added drop wise. After the reaction was completed (monitored by TLC), the solvent was evaporated under reduced pressure and the precipitate was recrystallized in ethanol. Yield 83%; white crystalline powder; mp: 125-127 ºC; IR (KBr disk): υ (cm^-1^) 1165, 1308 (SO_2_) 1710 (C=O); LC-MS (ESI) m/z: 276.7 (M+1, 100).

*6-(4-(Methylsulfonyl)phenyl)imidazo[2,1-b]thiazole (* ***5*** *)*

To 1 g of 3 (3.62 mmol) in ethanol, 0.38 g Na_2_CO_3_ (7.25 mmol) and 0.36 g 2-aminothiazole (3.4 mmol) were added and refluxed for 24 hours. The precipitate was filtered off and washed with water. Yield 70.5%; brown powder; mp: 145.5-147 ºC; IR (KBr disk): νcm^-1^ 1149, 1298 (SO_2_);^ 1^HNMR (CDCl_3_): δ ppm 3.08 (s, 3H, SO_2_CH_3_), 6.94 (d, 1H, imidazothiazole H_2_), 7.51 (d, 1H, imidazothiazole H_3_), 7.89-8.01 (m, 5H, imidazothiazole H_5_, 4-methylsulfonylphenyl H_2,_ H_3,_ H_5_ & H_6_, J = 6.9 Hz); LC-MS (ESI) m/z: 279.0 (M+1, 100). Anal. Calcd. for C_12_H_10_N_2_O_2_S_2_: C, 51.78; H, 3.62; N, 10.06. Found: C, 51.52; H, 3.85; N, 10.25.

*6-(4-(Methylsulfonyl)phenyl)imidazo[2,1-b]thiazole derivatives (* ***6a-g*** *)*

At 0 ºC, acetic acid, ~36% formalin solution (1.37 mmol) and appropriate aliphatic amine 1.37 mmol) were added in the flask. Then, **4** (0.3 g, 1.10 mmol) was added and the reaction mixture stirred at 50 ºC for reaction completion. The reaction mixture was cooled under 10 ºC and pH was adjusted to ~8-9 with 20% sodium hydroxide solution. The solid was filtered and washed with water. The crude was purified by chromatography to give compounds (**6a-g**) (Yield 50-70%).

*N,N-Dimethyl-1-(6-(4-(methylsulfonyl)phenyl)imidazo[2,1-b]thiazol-5-yl)methanamine (* ***6a*** *)*

Yield 61%; Yellow powder; mp: 123-124 ºC; IR (KBr): 1 ν (cm^-1^) 1156, 1316 (SO_2_); ^1^HNMR (CDCl_3_): δ ppm 2.28 (s, 6H, CH_3_), 3.08 (s, 3H, SO_2_CH_3_), 3.79 (s, 2H, -CH_2_-N), 6.85 (d, 1H, imidazothiazole H_2_, J = 4.4 Hz), 7.68 (d, 1H, imidazothiazole H_3_, J = 4.4 Hz), 7.98 (dd, 4H, 4-methylsulfonylphenyl H_2_, H_3_, H_5_ and H_6_, J = 6.6 Hz); LC-MS (ESI) m/z : 336.0 (M+1, 100). Anal. Calcd. for C_15_H_17_N_3_O_2_S_2_: C, 53.71; H, 5.11; N, 12.53. Found: C, 53.51; H, 5.35; N, 12.84.

*N-Ethyl-N-((6-(4-(methylsulfonyl)phenyl)imidazo[2,1-b]thiazol-5-yl)methyl)ethanamine (* ***6b*** *)*

Yield 55.1%; Yellow powder; mp: 132-135 ºC; IR (KBr): ν (cm^-1^) 1152,1304 (SO_2_); ^1^HNMR (CDCl_3_): δ ppm 1.04-1.07 (t, 6H, CH_3_, J = 7.1 Hz), 2.54-2.58 (q, 4H ,CH_2_-CH_3_, J = 7.1 Hz), 3.12 (s, 3H ,SO_2_CH_3_), 3.97 (s, 2H -CH_2_-N), 6.86 (d, 1H, imidazothiazole H_2_, J = 4.5 Hz), 7.81 (d, 1H, imidazothiazole H_3_, J = 4.5 Hz),8.01 (dd, 4H, 4-methylsulfonylphenyl H_2,_ H_3,_ H_5_ & H_6_, J=6.6 Hz); LC-MS (ESI) m/z : 364.0 (M+1, 100). Anal. Calcd. for C_17_H_21_N_3_O_2_S_2_: C, 56.17; H, 5.82; N, 11.56. Found: 56.35; H, 6.02; N, 11.71.

*N-((6-(4-(Methylsulfonyl)phenyl)imidazo[2,1-b]thiazol-5-yl)methyl)-N-propylpropan-1-amine (* ***6c*** *)*

Yield 54.9%; Yellow powder; mp: 165-167 ºC; IR (KBr): ν (cm^-1^) 1155,1307 (SO_2_); ^1^HNMR (CDCl_3_): δ ppm 0.84-0.87 (t, 6H, CH_3_, J = 7.3 Hz), 1.46-1.53 (m, 4H,-CH_2_-CH_3_), 2.41-2.43 (t,4H,-CH_2_-CH_2_, J = 7.1 Hz), 3.12 (s, 3H, SO_2_CH_3_), 3.96 (s, 2H, -CH_2_-N), 6.86 (d, 1H, imidazothiazole H_2_, J = 4.0 Hz), 7.76 (d, 1H, imidazothiazole H_3_, J = 4.0 Hz), 8.01 (dd, 4H, 4-methylsulfonylphenyl H_2_, H_3_, H_5_ and H_6_, J = 6.7 Hz) ; LC-MS (ESI) m/z : 392.1 (M+1, 100). Anal. Calcd. for C_19_H_25_N_3_O_2_S_2_: C, 58.28; H, 6.44; N, 10.73*.* Found: C, 58.33; H, 6.57; N, 10.98*.*

*6-(4-(Methylsulfonyl)phenyl)-5-(pyrrolidin-1-ylmethyl)imidazo[2,1-b]thiazole (* ***6d*** *)*

Yield 51%; Yellow powder; mp: 144-146 ºC; IR (KBr): ν (cm^-1^) 1147,1307 (SO_2_); ^1^HNMR (CDCl_3_): δ ppm 1.82 (m, 4H, CH_2_-), 2.57 (m, 4H, -N-CH_2_-), 3.11 (s, 3H, SO_2_CH_3_), 4.03 (s, 2H, -CH_2_-N), 6.87 (d, 1H, imidazothiazole H_2_, J = 4.2 Hz), 7.78 (d, 1H, imidazothiazole H_3_, J = 4.2 Hz), 8.00 (dd, 4H, 4-methylsulfonylphenyl H_2_, H_3_, H_5_ and H_6_, J = 6.8 Hz); LC-MS (ESI) m/z : 362.00 (M+1, 100). Anal. Calcd. for C_17_H_19_N_3_O_2_S_2_: C, 56.49; H, 5.30; N, 11.62. Found: C, 56.13; H, 5.51; N, 11.88.

*6-(4-(Methylsulfonyl)phenyl)-5-(piperidin-1-ylmethyl)imidazo[2,1-b]thiazole (* ***6e*** *)*

Yield 60%; Yellow powder; mp: 142.6-144 ºC; IR (KBr): ν (cm^-1^) 1151, 1309 (SO_2_); ^1^HNMR (CDCl_3_): δ ppm 1.50 (m, 2H, -CH_2_-),1.58-1.63 (m, 4H, -CH_2_-), 2.46 (s, 2H, -CH_2_-N), 3.12 (s, 3H, SO_2_CH_3_), 3.84 (t, 4H, N-CH_2_-), 6.87 (d, 1H, imidazothiazole H_2_, J = 4.4 Hz), 7.78 (d, 1H, imidazothiazole H_3_, J = 4.4 Hz), 8.02 (dd, 4H, 4-methylsulfonyl-phenyl H_2_, H_3_, H_5 _and H_6_, J = 6.7 Hz) ; LC-MS (ESI) m/z : 376 (M+1, 100). Anal. Calcd. for C_18_H_21_N_3_O_2_S_2_: C, 57.58; H, 5.64; N, 11.19. Found: C, 57.78; H, 5.91; N, 11.26. 

*4-((6-(4-(Methylsulfonyl)phenyl)imidazo[2,1-b]thiazol-5-yl)methyl)morpholine (* ***6f*** *)*

Yield 69.5%; Yellow powder; mp: 203-206 ºC; IR (KBr): ν (cm^-1^) 1173, 1331 (SO_2_); ^1^HNMR (CDCl_3_): δ ppm 2.49 (t, 4H, -N-CH_2_-, *J = *5.0 Hz), 3.08 (s, 3H, SO_2_CH_3_), 3.69-3.71 (t, 4H, -CH_2_-O, J = 4.2), 3.86 (s, 2H, -CH_2_-N), 6.87 (d, 1H, imidazothiazole H_2_, J = 4.4 Hz), 7.70 (d,1H, imidazothiazole H_3_, J = 4.4 Hz), 7.98 (dd, 4H, 4-methylsulfonylphenyl H_2_, H_3_, H_5_ and H_6_, J = 6.9 Hz ); LC-MS (ESI) m/z : 378.1 (M+1, 100). Anal. Calcd. for C_17_H_19_N_3_O_3_S_2_: C, 54.09; H, 5.07; N, 11.13. Found: C, 54.23; H, 5.29; N, 11.33.

*2,2′-(((6-(4-(Methylsulfonyl)phenyl)imidazo[2,1-b]thiazol-5-yl)methyl)azanediyl)bis(ethan-1-ol) (* ***6g*** *)*

Yield 65%; white powder; mp: 168.8-170 ºC; IR (KBr): ν (cm^-1^) 1170, 1331 (SO_2_), 3417 (OH) ; ^1^HNMR (CDCl_3_): δ ppm 2.13-2.14 (bs, 2H, O-H), 2.74-2.76 (t, 4H, -N-CH_2_-, J = 5.0 Hz), 3.13 (s, 3H, SO_2_CH_3_), 3.66-3.68 (t, 4H, O-CH_2_-, J = 5.0 Hz), 4.18 (s, 2H, -CH_2_-N), 6.91 (d, 1H, imidazothiazole H_2_, J = 4.4 Hz), 7.91 (d, 1H, imidazothiazole H_3, _J = 4.4 Hz), 7.96-7.98 (d, 2H, 4-methylsulfonylphenyl H_2_ and H_6,_ J = 8.3 Hz); 8.02 (d, 2H, 4-methylsulfonylphenyl H_3_ and H_5,_* J *= 8.3Hz); LC-MS (ESI) m/z: 396.1 (M+1,100). Anal. Calcd. for C_17_H_21_N_3_O_4_S_2_: C, 51.63; H, 5.35; N, 10.62. Found: C, 51.77; H, 5.65; N, 10.82.

*Molecular modeling studies*

Docking studies were implemented using AutoDock software Version 4.2. The coordinates of the X-ray crystal structure of SC-558 as known selective COX-2 inhibitor bound to the murine COX-2 enzyme was obtained from the RCSB Protein Data Bank (6COX) and hydrogens were added. All the ligand molecules were built by the Builder module and were energy minimized for 1000 iterations reaching a convergence of 0.01 kcal/mol Å. The energy minimized ligands were superimposed on SC-558 in the PDB file of 6COX after which SC-558 was deleted. Searching for the desired binding configuration between the small flexible ligands and the rigid protein is the purpose of docking study. For efficiency, protein residues with atoms greater than 6.0 Å from the docking box were removed. The quality of the docked structures was evaluated by measuring the intermolecular energy of the ligand-enzyme assembly (32, 33).

*In-vitro cyclooxygenase (COX) inhibition assay*

Enzyme chemiluminescent kit (Cayman chemical, MI, USA) was used to evaluate inhibition activities of synthesized compounds based on our previous reported procedure (34). The Cayman chemical chemiluminescent COX (ovine) inhibitor screening assay employs the heme-catalyzed hydroperoxidase activity of ovine cyclooxygenases to produce luminescence in the presence of a cyclic naphthalene hydrazide and the substrate arachidonic acid. Arachidonate-induced luminescence was shown to be an index of real-time catalytic activity and demonstrated the turnover inactivation of the enzyme. COX inhibitory activity, measured by luminescence, by a variety of selective and nonselective inhibitors showed potencies similar to those observed with other *in-vitro* and whole cell methods.

## Results and Discussion

*Chemistry*

**Scheme 1:**
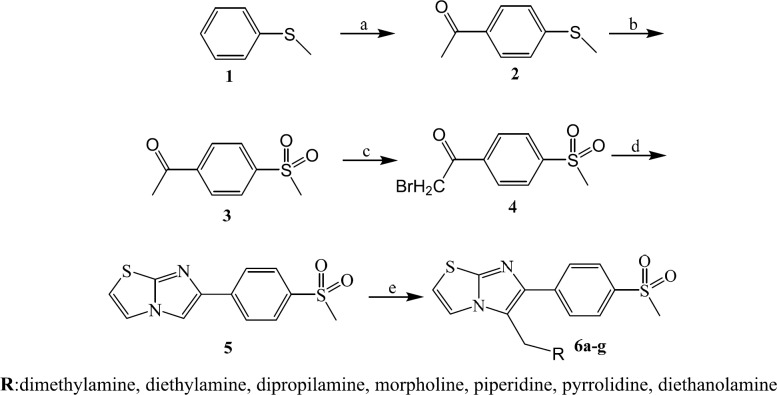
Reagents and conditions: (a) AlCl_3_, CH_3_COCl, CHCl_3_, 25 °C , 2h (b) Oxone, THF, water, 25 °C, 2h (c) Br_2_ , CHCl_3_, 25 °C (d) Ethanol, Na_2_CO_3_, 2-aminothiazole, reflux (e) acetic acid, formalin, different aliphatic amines

The synthesis of 6-(4-(methylsulfonyl)phenyl)imidazo[2,1-b]thiazole derivatives was carried out according to Scheme 1 (31). Starting from thioanisole (**1**), acetyl chloride and aluminium chloride through a Friedel-crafts reaction, 4-(methylthio)acetophenone was obtained (**2**). (Methylsulfonyl)acetophenone (**3**) was prepared using oxone in THF/water. The bromination of compound (**3**) via bromine in CHCl_3 _at room temperature afforded α-bromo-4-(methylsulfonyl)acetophenone (**4**). Compound (**4**) was treated with 2-aminothiazole to yield 6-(4-(methylsulfonyl)phenyl)imidazo[2,1-b]thiazole (**5**). The target molecules (**6a-g**) were synthesized in presence of appropriate aliphatic amines, formalin in acetic acid. The purity of all products was determined by thin layer chromatography using different polarity solvent systems. The structures of the synthesized compounds were confirmed by IR, ^1^HNMR, and ESI-MS.

*Docking study*

**Figure 2:**
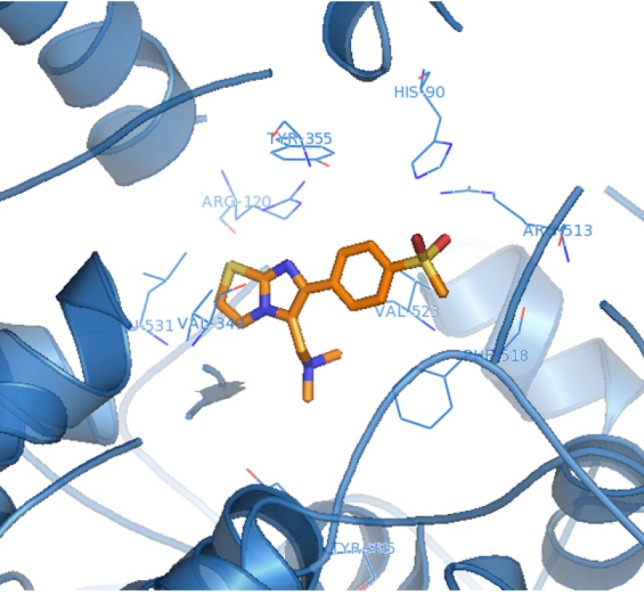
Docking N,N-dimethyl-1-(6-(4-(methylsulfonyl)phenyl)imidazo[2,1-b]thiazol-5- yl)methanamine (**6a**) in the active site of murine COX-2. Hydrogen atoms have been removed to improve clarity

**Figure 3:**
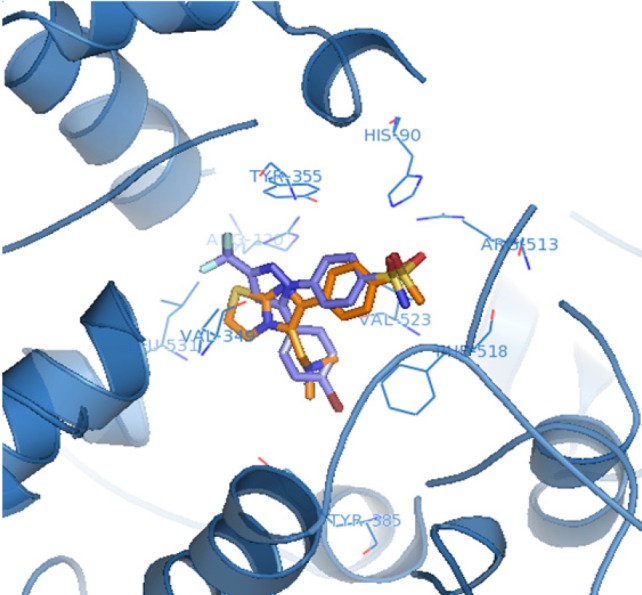
Superimposition of compound (**6a**) on the SC558 molecule

The orientation and binding interactions of the most selective COX-2 inhibitor (**6f**) within the COX-2 binding site were evaluated by a docking experiment. Results obtained from docking procedure of **6a** as selective and most potent COX-2 inhibitor among the synthesized compounds into the COX-2 binding site (Figure 2) showed that the *para*-SO_2_Me substituent inserts into the secondary pocket present in COX-2 (Arg^513^, Phe^518^, Val^523^). One of O-atoms of *para*-SO_2_Me forms a hydrogen bonding interaction with the amino group of Arg^513^ (distance = 3.27 Å). Additional docking study showed that **6a** and SC558 were superimposed tightly in COX active site (Figure 3).

*Enzyme inhibitory activity*

The ability of 6-(4-(methylsulfonyl)phenyl)imidazo[2,1-b]thiazole derivatives to inhibit COX-1 and COX-2 was measured using chemiluminescent enzyme assay to investigate the effect of different substituents on COX-2 selectivity and potency (Table 1). According to *in-vitro* COX-1/COX-2 inhibition data, all compounds (**6a-g**) were selective inhibitors of COX-2 isoenzyme with IC_50_ values in the highly potent 0.08-0.16 µM range. These results indicated that both potency and selectivity of COX-2 inhibitory activity were affected by the type and size of amine on C-5 of imidazo[2,1-b]thiazole ring. Based on these results N,N-dimethyl-1-(6-(4-(methylsulfonyl)phenyl)imidazo[2,1-b]thiazol-5-yl)methanamine (6a) was proved to be the most potent (IC_50 _= 0.08 µM) COX-2 inhibitor among the synthesized compounds. These results showed that replacement of dimethylamino group with larger dialkylamino substituents such as diethylamino (**6b**) or dipropylamino (**6c**) decreased both potency and selectivity for COX-2 inhibitory activity. Our results also indicated that compounds having cyclic amino groups such as pyrrolidine (**6d**), piperidine (**6e**) or morpholine (**6f**) showed moderate to high COX-2 selectivity. However, compound **6f** having morpholine group had higher potency and selectivity for COX-2 inhibitory activity compared with compound **6e** possessing piperidine substituent which might be explained by the ability of morpholine for hydrogen binding with COX-2 active site. Similarly, compound 6g containing diethanolamine with hydrogen binding potential showed high COX-2 inhibitory potency and selectivity. These findings confirm that synthesized compounds should inhibit the biosynthesis of prostaglandins through the cyclooxygenase pathway at sites of inflammation. 

**Table 1:** *In-vitro* COX-1 and COX-2 enzyme inhibition assay data

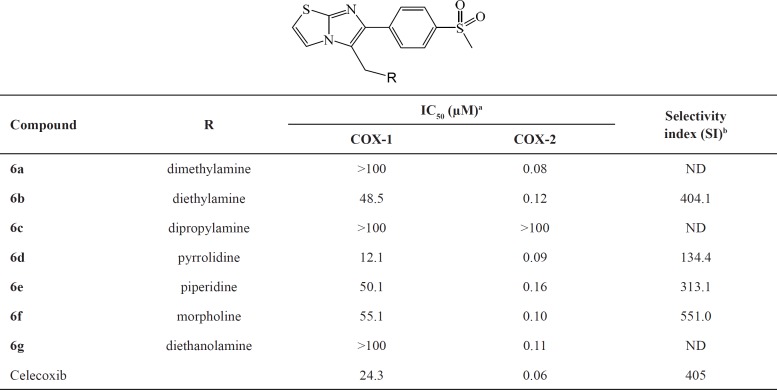

a Values are means of two determinations acquired using an ovine COX-1/COX-2 assay kit and the deviation from the mean is <5% of the mean value.

b *In-vitro *COX-2 selectivity index (COX-1 IC_50_/COX-2 IC_50_).

## Conclusion

The results of this investigation indicated that (i) 6-(4-(methylsulfonyl)phenyl)imidazo[2,1-b]thiazole is a suitable scaffold to design COX-2 inhibitors, (ii) COX-1/-2 inhibition is sensitive to the type of substituent at C-5 of imidazo[2,1-b]thiazole ring, (iii) N,N-dimethyl-1-(6-(4-(methylsulfonyl)phenyl)imidazo[2,1-b]thiazol-5-yl)methanamine (**6a**) exhibited highly COX-2 inhibitory potency.
